# Comparison of COVID-19 Hospitalization and Death Between Solid Organ Transplant Recipients and the General Population in Canada, 2020–2022

**DOI:** 10.1097/TXD.0000000000001670

**Published:** 2024-06-26

**Authors:** Kyla L. Naylor, Gregory A. Knoll, Darin Treleaven, Yuguang Kang, Amit X. Garg, Kathryn Stirling, S. Joseph Kim

**Affiliations:** 1 ICES, ON, Canada.; 2 Department of Epidemiology & Biostatistics, Western University, London, ON, Canada.; 3 Lawson Health Research Institute, London Health Sciences Centre, London, ON, Canada.; 4 Department of Medicine (Nephrology), University of Ottawa and the Ottawa Hospital Research Institute, Ottawa, ON, Canada.; 5 Trillium Gift of Life Network, Ontario Health, ON, Canada.; 6 Division of Nephrology, University Health Network, University of Toronto, Toronto, ON, Canada.

## Abstract

**Background.:**

Solid organ transplant recipients have a high risk of severe outcomes from SARS-CoV-2 infection. A comprehensive understanding of the impact of the COVID-19 pandemic across multiple waves in the solid organ transplant population and how this compares to the general population is limited. We conducted a population-based cohort study using linked administrative healthcare databases from Ontario, Canada to answer this question.

**Methods.:**

We included 15 306 solid organ transplant recipients and 12 160 904 individuals from the general population. Our primary outcome was the rate (per 100 person-years) of severe COVID-19 (ie, hospitalization or death with a positive SARS-CoV-2 test) occurring between January 25, 2020, and November 30, 2022.

**Results.:**

Compared with the general population, solid organ transplant recipients had almost a 6 times higher rate of severe COVID-19 (20.39 versus 3.44 per 100 person-years), with almost 5.5 times as high a rate of death alone (4.19 versus 0.77 per 100 person-years). Transplant recipients with severe COVID-19 were substantially younger (60.1 versus 66.5 y) and had more comorbidities. The rate of severe COVID-19 declined over time in the solid organ transplant population, with an incidence rate of 41.25 per 100 person-years in the first wave (January 25, 2020, to August 31, 2020) and 18.41 in the seventh wave (June 19, 2022, to November 30, 2022, Omicron era).

**Conclusions.:**

Solid organ transplant recipients remain at high risk of severe outcomes when they are infected with SARS-CoV-2. Resources and strategies to mitigate the impact of SARS-CoV-2 exposure are needed in this vulnerable patient population.

The number of individuals living with a solid organ transplant continues to increase worldwide, with >144 000 transplants performed in 2021.^1^ These individuals often have multiple comorbidities (eg, diabetes), require immunosuppressive medications, and have altered immune response to vaccines (eg, lower protection against COVID-19 after vaccination).^2-4^ There is concern about the ongoing impact of COVID-19 infection in solid organ transplant recipients.

Numerous studies have examined the impact of COVID-19 in solid organ transplant recipients.^5-17^ These studies have consistently found that solid organ transplant recipients have a high rate of death and hospitalization from COVID-19. However, few studies have provided a comprehensive look at COVID-19 and its associated outcomes in all solid organ transplant types, across multiple waves of the COVID-19 pandemic, including 1-y into the Omicron era when patients had access to both vaccination and therapeutics. Furthermore, few studies have provided a direct comparison of COVID-19 outcomes between solid organ transplant recipients and the general population.

Understanding the impact of the COVID-19 pandemic on solid organ transplant recipients is needed to target public health campaigns (eg, vaccination), guide resource planning (eg, hospital resources), influence funding decisions, and promote the development of strategies to improve outcomes in this vulnerable population.^18^ Therefore, we conducted this study to comprehensively examine the impact of the COVID-19 pandemic (eg, hospitalization and death with a positive SARS-CoV-2 test) in solid organ transplant recipients when compared with the general population across multiple waves of the COVID-19 pandemic.

## MATERIALS AND METHODS

### Design and Setting

We conducted a population-based cohort study using administrative healthcare databases. These datasets were linked using unique encoded identifiers and analyzed at ICES Western in Ontario, Canada (ices.on.ca). ICES is an independent, nonprofit research institute whose legal status under Ontario’s health information privacy law allows it to collect and analyze healthcare and demographic data, without consent, for health system evaluation and improvement. The use of data in this project was authorized under section 45 of Ontario’s Personal Health Information Protection Act, which does not require review by a Research Ethics Board. We reported this study following the REporting of studies Conducted using Observational Routinely-collected health Data statement (**Table S1, SDC**, http://links.lww.com/TXD/A675).^19^

### Data Sources

To identify solid organ transplant recipients, we used the Canadian Organ Replacement Register, which provides data on all Canadian solid organ transplant recipients. To supplement this information, we used the Ontario Health Insurance Plan (OHIP), which contains information on physician diagnostic and billing codes. To identify the general population cohort, we used the Registered Persons Database, which contains information on vital status and demographics. We obtained information on diagnostic and procedural codes occurring during a hospitalization from the Canadian Institute for Health Information Discharge Abstract Database.

We used the ICES-derived COVID-19 Integrated Testing Dataset to identify individuals who tested positive for SARS-CoV-2. This dataset is derived from 3 data sources, the Ontario Laboratories Information System, distributed testing data from laboratories within the COVID-19 diagnostic network, and the Case and Contact Management System (CCM). These datasets provide all available COVID-19 reverse transcription polymerase chain reaction (RT-PCR) results in Ontario. In combination with the COVID-19 Integrated Testing Dataset, we used CCM (ie, central data repository for Ontario COVID-19 reporting) to capture a hospitalization or death with a positive SARS-CoV-2 test. To capture information on COVID-19 vaccinations, we used COVaxON, which is Ontario’s centralized vaccination system. **Table S2 (SDC**, http://links.lww.com/TXD/A675) provides further details on the databases and coding definitions.

### Study Populations

#### Solid Organ Transplant Recipients

We included adults (ie, aged ≥18 y) with a solid organ transplant (ie, kidney, liver, heart, lung, pancreas [could include simultaneous kidney-pancreas], and other multiorgan transplants) between January 25, 2020 (first recorded date of a positive SARS-CoV-2 infection in Ontario) and September 30, 2022. The cohort entry date (index date) was January 25, 2020, for solid organ transplant recipients who received a transplant on or before this date and was the date of transplant for individuals who received a transplant between January 26, 2020, and September 30, 2022. We excluded individuals who died on or before their index date. For recipients who entered the cohort on January 25, 2020, we excluded kidney transplant recipients with evidence of graft failure (ie, evidence of maintenance dialysis) and recipients who did not have evidence of OHIP coverage in the quarter closest to the index date. Lastly, for recipients entering the cohort after January 25, 2020, we excluded individuals who had a positive SARS-CoV-2 test in the 90 d before their transplant date; it is uncommon for a new infection to occur in this time frame.^20^

#### General Population

To provide context into the burden of COVID-19 in solid organ transplant recipients, we included adults (ie, aged ≥18 y) from the general population. We randomly assigned a cohort entry date to all adults in Ontario, Canada (>14 000 000 residents), by randomly sampling index dates (with replacement) from the recipient group. We excluded individuals who died on or before the cohort entry date, had no evidence of OHIP coverage in the previous quarter, had a positive SARS-CoV-2 test in the 90 d before cohort entry, and individuals who had previously received a solid organ transplant.

### Outcomes

Our primary outcome was severe COVID-19 (ie, hospitalization or death with a positive SARS-CoV-2 test) captured in CCM. To supplement CCM, we used information from Canadian Institute for Health Information to capture hospitalizations defined as a positive SARS-CoV-2 test occurring in the 14 d before or 3 d after a hospital admission. We used the Registered Persons Database to supplement CCM death information, which was defined as a positive SARS-CoV-2 positive test in the 30 d before death. We followed patients from their cohort entry to a maximum follow-up date of November 30, 2022.

Our secondary outcomes, included hospitalization with a positive SARS-CoV-2 test or death with a positive SARS-CoV-2 test examined separately, length of stay for a hospital admission with a positive SARS-CoV-2 test, admission to the intensive care unit with a positive SARS-Cov-2 test, RT-PCR-confirmed SARS-CoV-2 infection, and RT-PCR SARS-CoV-2 testing (ie, inclusion of negative and positive test results).

In Ontario, restrictions to laboratory-based RT-PCR testing were introduced on December 30, 2021. Only high-risk individuals (excluding solid organ transplant recipients) or individuals working in high-risk settings with symptoms were eligible for testing. However, many solid organ transplant recipients could still access confirmatory PCR tests from transplant centers and it is standard to assess all individuals for COVID-19 infection (ie, infection control measure) upon hospital admission.

### Statistical Analysis

We presented baseline characteristics as means (± SD) or medians (25th–75th percentile) for continuous variables and as frequencies (proportions) for categorical variables. We compared baseline characteristics between solid organ transplant recipients and the general population using standardized differences, with >10% considered a notable difference.^21^

For the primary outcome, we reported the frequency and percentage of events, and the event rate per 100 person-years (95% confidence interval [CI]). We censored individuals at non-COVID death and end of follow-up (November 30, 2022). To compare the event rates in the solid organ transplant population to the general population, we calculated the incidence rate ratio (IRR) (95% CI). We used the Mann-Whitney *U* test to compare the median length of hospital stay with a positive SARS-CoV-2 test between solid organ transplant recipients and the general population. We defined statistical significance as a 2-sided *P* value <0.05. We did not adjust CI widths for multiple testing. All analyses were conducted using SAS, Version 9.4 (SAS Institute, Cary, NC).

### Additional Analyses

For the primary outcome, we reported the IRRs comparing solid organ transplant recipients to the general population in the following subgroups: age (18 to <40, 40 to <60, 60 to <70, 70 to <80, 80+ y), dominant COVID-19 variant of concern in Ontario (earlier variant/nonvariant of concern: Before January 17, 2021; Alpha: January 17, 2021, to June 12, 2021; Delta: June 13, 2021, to December 1, 2021; Omicron: December 1, 2021, to November 30, 2022, with BA.1 dominating until February 2022, BA.2 until May 2022, and BA.4/5 until the end of the study period [November 30, 2022]),^22^ prevalent versus incident cohort (prevalent cohort: index date January 25, 2020, versus incident cohort: index date between January 26, 2020, and September 20, 2022), and 7 waves of the COVID-19 pandemic, that is, wave 1 (January 25, 2020, to August 31, 2020), wave 2 (September 1, 2020, to February 28, 2021), wave 3 (March 1, 2021, to July 31, 2021), wave 4 (August 1, 2021, to December 14, 2021), wave 5 (December 15, 2021, to February 28, 2022), wave 6 (March 1, 2022, to June 18, 2022), and wave 7 (June 19, 2022, to November 30, 2022). Lastly, we restricted to individuals who survived 90 d after their initial SARS-CoV-2 infection and reported on the frequency (proportion) of individuals who had at least 1 reinfection during follow-up, which we defined as a subsequent SARS-CoV-2 infection >90 d after the first infection.

## RESULTS

We included 15 306 solid organ transplant recipients and 12 160 904 individuals from the general population (**Figures S1** and **S2, SDC**, http://links.lww.com/TXD/A675). Solid organ transplant types included kidney (n = 8853 [57.8%]), liver (n = 3510 [22.9%]), lung (n = 1213 [7.9%]), heart (n = 973 [6.4%]), pancreas (n = 636 [4.2%]), and multiorgan (n = 121 [0.8%]) transplants. Table 1 displays baseline characteristics for the solid organ transplant and general populations. Compared with the general population, solid organ transplant recipients were significantly older (57 versus 49 y), less likely to be female (37.5% versus 51.1%), and had significantly more comorbidities (eg, congestive heart failure: 19.9% versus 2.3%; chronic respiratory disease: 32.2% versus 20.4%; and diabetes: 46.1% versus 11.9%).

**TABLE 1.** Baseline characteristics for solid organ transplant recipients and the general population

Characteristics	Solid organ transplant recipient (n = 15 306)	General population (n = 12 160 904)	Standardized difference, %^*a*^
Age, y	57.0 ± 14.2	48.8 ± 18.5	**50**
18 to <40	2038 (13.3)	4 377 865 (36.0)	**55**
40 to <60	5694 (37.2)	4 127 384 (33.9)	7
60 to <70	4714 (30.8)	1 807 088 (14.9)	**39**
70 to <80	2487 (16.2)	1 158 063 (9.5)	**20**
≥80	373 (2.4)	690 504 (5.7)	**17**
Female	5740 (37.5)	6 210 539 (51.1)	**28**
Neighborhood income quintile^*b*^			
1 (Lowest)	3240 (21.2)	2 407 852 (19.8)	3
2	3129 (20.4)	2 420 490 (19.9)	1
3	3122 (20.4)	2 475 783 (20.4)	0
4	2939 (19.2)	2 423 343 (19.9)	2
5 (Highest)	2876 (18.8)	2 433 436 (20.0)	3
Rural^*c*^	1704 (11.1)	1 227 810 (10.1)	3
Time since transplant^*d*^, y	7 (3–13)		
Long-term care status	70 (0.5)	78 327 (0.6)	1
Charlson Comorbidity Index^*e*^	0 (0–2)	0 (0–0)	**109**
0	8590 (56.1)	11 767 077 (96.8)	**109**
1	757 (4.9)	163 363 (1.3)	**21**
2	2535 (16.6)	115 818 (1)	**57**
3	1147 (7.5)	58 548 (0.5)	**36**
≥4	2277 (14.0)	56 098 (0.5)	**56**
Congestive heart failure	3053 (19.9)	278 912 (2.3)	**58**
Hypertension	12 305 (80.4)	3 141 692 (25.8)	**131**
Diabetes	7063 (46.1)	1 451 836 (11.9)	**81**
Dementia	308 (2.0)	200 033 (1.6)	3
Stroke (excluding transient ischemic attack)	1047 (6.8)	235 962 (1.9)	**24**
Myocardial infarction	521 (3.4)	96 843 (0.8)	**18**
Chronic respiratory disease^*f*^	4932 (32.2)	2 478 253 (20.4)	**27**
Chronic kidney disease	10 777 (70.4)	287 559 (2.4)	**200**
Major cancer^*g*^	2734 (17.9)	596 959 (4.9)	**42**
Chronic liver disease	9248 (60.4)	3 415 315 (28.1)	**69**

Data are presented as n (%), mean ± SD, or median (25th–75th percentile).

^*a*^Standardized differences measure the difference between groups divided by the pooled SD; a value >10% is a meaningful difference between the solid organ transplant and general population groups. Bold standard differences denote a meaningful difference.

^*b*^Income presented as quintiles of average neighborhood income (<0.5% missing and was imputed as quintile 3).

^*c*^Rural is defined as living in an area with a population < 10 000 (<0.5% missing and was imputed as urban).

^*d*^Time since transplant was only measured in solid organ transplant recipients who entered the cohort on January 25, 2020 (ie, prevalent solid organ transplant recipients, n = 12 708).

^*e*^The Charlson Comorbidity Index is a weighted predictive tool that uses International Classification of Diseases codes to predict the risk of mortality and increased resource use for patients.

^*f*^Chronic respiratory disease defined as asthma or chronic obstructive pulmonary disease.

^*g*^Major cancer is defined as a composite of lung/bronchi, colon/rectum, breast, pancreas, prostate, leukemia, non-Hodgkin lymphoma, liver, ovarian, and esophageal. Evidence of major cancer was assessed in the 5 y before index date.

Over a median follow-up of 2.8 y (2.1–2.8 y), 3385 (22.1%) solid organ transplant recipients tested positive for SARS-CoV-2, of which 1563 (46.2%) had severe COVID-19. In the general population, 1 094 440 (9.0%) tested positive for SARS-CoV-2, of which 98 927 (9.0%) had severe COVID-19 over a median follow-up of 2.8 y (2.8–2.8 y). During follow-up, 1462 (9.6%) solid organ transplant recipients had a non-COVID-19 death compared with 275 576 (2.3%) individuals in the general population.

Table 2 shows the incidence rates and IRRs for severe COVID-19 and the rate of hospitalization and death examined separately among solid organ transplant recipients and the general population. When compared with the general population, solid organ transplant recipients had almost 6 times as high a rate of severe COVID-19 (IRR, 5.92; 95% CI, 5.63-6.23). When examining the rate of severe COVID-19 by solid organ transplant type, we found that multiorgan transplant recipients and lung transplant recipients had the highest rate (30.15 per 100 person-years in multiorgan transplant recipients and 29.50 in lung transplant recipients). When just examining hospitalization with a positive SARS-CoV-2 test, we found solid organ transplant recipients had >6 times as high a hospitalization rate when compared with the general population (IRR, 6.32; 95% CI, 6.00-6.64). When examining death with a positive SARS-CoV-2 test, we found solid organ transplant recipients had almost 5.5 times as high a rate of death (IRR, 5.47; 95% CI, 4.93-6.07) compared with the general population with a rate of 4.19 (95% CI, 3.78-4.64) per 100 person-years in solid organ transplant recipients and 0.77 (95% CI, 0.76-0.78) in the general population.

**TABLE 2.** Incidence rate and incidence rate ratio of severe COVID-19 (ie, hospitalization or death with a positive SARS-CoV-2 test) and hospitalization and death with a positive SARS-CoV-2 test examined separately among solid organ transplant recipients and the general population who tested positive for SARS-CoV-2 (January 25, 2020, to November 30, 2022)

Population^*a*^	Number of events (%)	Rate per 100 person-years (95% CI)	Incidence rate ratio (95% CI)	Rate per 100 person-years (95% CI)	Incidence rate ratio (95% CI)	Rate per 100 person-years (95% CI)	Incidence rate ratio (95% CI)
	Hospitalization or death with a positive SARS-CoV-2 test^*b*^			Hospitalization with a positive SARS-CoV-2 test		Death with a positive SARS-CoV-2 test	
Solid organ transplant recipients (n = 3385)	1563 (46.2)	20.39 (19.40-21.42)	5.92 (5.63-6.23)	19.96 (18.98-20.98)	6.32 (6.00-6.64)	4.19 (3.78-4.64)	5.47 (4.93-6.07)
Kidney transplant (n = 2130)	1008 (47.3)	20.85 (19.60-22.18)	6.06 (5.69-6.44)	20.43 (19.20-21.75)	6.47 (6.07-6.89)	4.63 (4.09-5.24)	6.05 (5.35-6.85)
Liver transplant (n = 529)	159 (30.1)	12.90 (11.04-15.07)	3.75 (3.21-4.38)	12.33 (10.52-14.46)	3.90 (3.33-4.58)	2.47 (1.76-3.48)	3.23 (2.30-4.54)
Heart transplant (n = 215)	100 (46.5)	19.77 (16.25-24.05)	5.74 (4.72-6.99)	19.18 (15.71-23.40)	6.07 (4.97-7.41)	2.85 (1.74-4.64)	3.72 (2.28-6.07)
Lung transplant (n = 324)	200 (61.7)	29.50 (25.68-33.88)	8.57 (7.46-9.84)	29.20 (25.41-33.57)	9.24 (8.04-10.63)	5.80 (4.33-7.77)	7.58 (5.66-10.15)
Pancreas transplant (n = 161)	80 (49.7)	22.10 (17.75-27.51)	6.42 (5.16-7.99)	21.82 (17.50-27.21)	6.91 (5.54-8.61)	2.88 (1.71-4.87)^*c*^	3.77 (2.23-6.36)^*c*^
Multiorgan transplant (n = 26)	16 (61.5)	30.15 (18.47-49.21)	8.76 (5.36-14.30)	30.15 (18.47-49.21)	9.54 (5.85-15.58)		
General population (n = 1 094 440)	98 927 (9.0)	3.44 (3.42-3.46)		3.16 (3.14-3.18)		0.77 (0.76-0.78)	

^*a*^The denominator is restricted to individuals who tested positive for SARS-CoV-2.

^*b*^A small proportion of hospitalizations or deaths with a positive SARS-CoV-2 test were associated with a reinfection (18 [1.2%] in the solid organ transplant population and 1968 [2.0%] in the general population).

^*c*^In accordance with ICES privacy policies, cell sizes ≤5 cannot be reported; this includes being able to calculate small cells based on other information provided. Therefore, the incidence rate and incidence rate ratio for death with a positive SARS-CoV-2 test was combined for the pancreas and multiorgan transplant groups.

CI, confidence interval.

Table 3 demonstrates baseline characteristics among solid organ transplant recipients who had severe COVID-19 compared with the general population. In brief, solid organ transplant recipients with severe COVID-19 were substantially younger (61.9 versus 68.2 y) with only 5.2% aged ≥80 y compared with 34.4% of the general population. The general population had a substantially higher proportion of individuals residing in a long-term care home compared with solid organ transplant recipients (9.6% versus 1.5%). Solid organ transplant recipients with severe COVID-19 generally had substantially more comorbidities compared with the general population. For example, 65.6% of solid organ transplant recipients had diabetes and 92.5% had hypertension, compared with 37.6% and 65.3% in the general population, respectively. When examining COVID-19-specific characteristics, solid organ transplant recipients with severe COVID-19, were substantially less likely to be unvaccinated compared with the general population (55.9% versus 63.8%) and more likely to have ≥3 doses of the COVID-19 vaccine (31.8% versus 21.6%). With regards to the dominant variant of concern, in solid organ transplant recipients 76.8% of all severe COVID-19 cases occurred during Omicron compared with 61.2% in the general population.

**TABLE 3.** Baseline characteristics for solid organ transplant recipients and the general population who were hospitalized or died with a positive SARS-CoV-2 test^*a*^

Characteristic	Solid organ transplant recipient with a hospitalization or death with a positive SARS-CoV-2 test (n = 1563)	General population with a hospitalization or death with a positive SARS-CoV-2 test (n = 98 927)	Standardized difference, %^*b*^
Age, y	61.9 ± 13.3	68.2 ± 19.4	**38**
18 to <40	122 (7.8)	11 520 (11.6)	**13**
40 to <60	428 (27.4)	17 673 (17.9)	**23**
60 to <70	522 (33.4)	15 555 (15.7)	**42**
70 to <80	409 (26.2)	20 161 (20.4)	**14**
80+	82 (5.2)	34 018 (34.4)	**79**
Female	589 (37.7)	48 770 (49.3)	**24**
Neighborhood income quintile^*c*^			
1 (Lowest)	405 (25.9)	28 508 (28.8)	7
2	341 (21.8)	21 853 (22.1)	1
3	334 (21.4)	18 700 (18.9)	6
4	249 (15.9)	16 148 (16.3)	1
5 (Highest)	234 (15.0)	13 718 (13.9)	3
Rural^*d*^	126 (8.1)	8386 (8.5)	1
Long-term care status	24 (1.5)	9468 (9.6)	**36**
Charlson Comorbidity Index^*e*^			
0	716 (45.8)	70 913 (71.7)	**55**
1	64 (4.1)	7906 (8.0)	**16**
2	262 (16.8)	7155 (7.2)	**30**
3	131 (8.4)	5492 (5.6)	**11**
≥4	390 (25.0)	7461 (7.5)	**49**
Congestive heart failure	543 (34.7)	19 751 (20.0)	**33**
Hypertension	1445 (92.5)	64 587 (65.3)	**71**
Diabetes	1025 (65.6)	37 192 (37.6)	**58**
Dementia	84 (5.4)	18 767 (19.0)	**42**
Stroke (excluding transient ischemic attack)	151 (9.7)	12 363 (12.5)	9
Myocardial infarction	84 (5.4)	4106 (4.2)	9
Chronic respiratory disease^*f*^	654 (41.8)	35 749 (36.1)	**12**
Chronic kidney disease	1353 (86.6)	18 520 (18.7)	**185**
Major cancer^*g*^	278 (17.8)	15 852 (16.0)	5
Chronic liver disease	928 (59.4)	36 575 (37.0)	**46**
COVID-19-specific characteristics			
COVID-19 vaccine doses^*h*^			
Unvaccinated	873 (55.9)	63 127 (63.8)	**16**
1 dose	22 (1.4)	2179 (2.2)	6
2 dose	171 (10.9)	12 204 (12.3)	4
≥3 doses	497 (31.8)	21 417 (21.6)	**23**
Time since last vaccination to testing positive, d^*h*^	144 (76–241)	186 (10–269)	**25**
Dominant variant of concern^*h*^			
Earlier variant/non-variant of concern (before January 17, 2021)	129 (8.3)	15 466 (15.6)	**23**
Alpha (January 17, 2021, to June 12, 2021)	179 (11.5)	18 169 (18.4)	**19**
Delta (June 13, 2021, to December 1, 2021)	55 (3.5)	4753 (4.8)	7
Omicron (December 1, 2021 onwards)	1200 (76.8)	60 539 (61.2)	**34**

Data are presented as n (%), mean ± SD, or median (25th–75th percentile).

^*a*^The date of hospital admission or death associated with a SARS-CoV-2 positive test was used as the reference date to pull these baseline characteristics.

^*b*^Standardized differences measure the difference between groups divided by the pooled SD; a value >10% is a meaningful difference between the solid organ transplant and general population groups. Bold standard differences denote a meaningful difference.

^*c*^Income presented as quintiles of average neighborhood income.

^*d*^Rural is defined as living in an area with a population < 10 000.

^*e*^The Charlson Comorbidity Index is a weighted predictive tool that uses International Classification of Diseases codes to predict risk of mortality and increased resource use for patients.

^*f*^Chronic respiratory disease defined as asthma or chronic obstructive pulmonary disease.

^*g*^Major cancer is defined as a composite of lung/bronchi, colon/rectum, breast, pancreas, prostate, leukemia, non-Hodgkin lymphoma, liver, ovarian, and esophageal. Evidence of major cancer was assessed in the 5 y before index date.

^*h*^Vaccination status was taken at the time of hospitalization or death. For example, if an individual only had evidence of 1 vaccine dose before their hospital admission or death associated with a SARS-CoV-2 positive test, they would be classified as having 1 dose.

^*i*^Categories selected based on the dominant variant circulating in the Ontario, Canada population at the time of the individual’s positive SARS-CoV-2 test.

**FIGURE 1.**
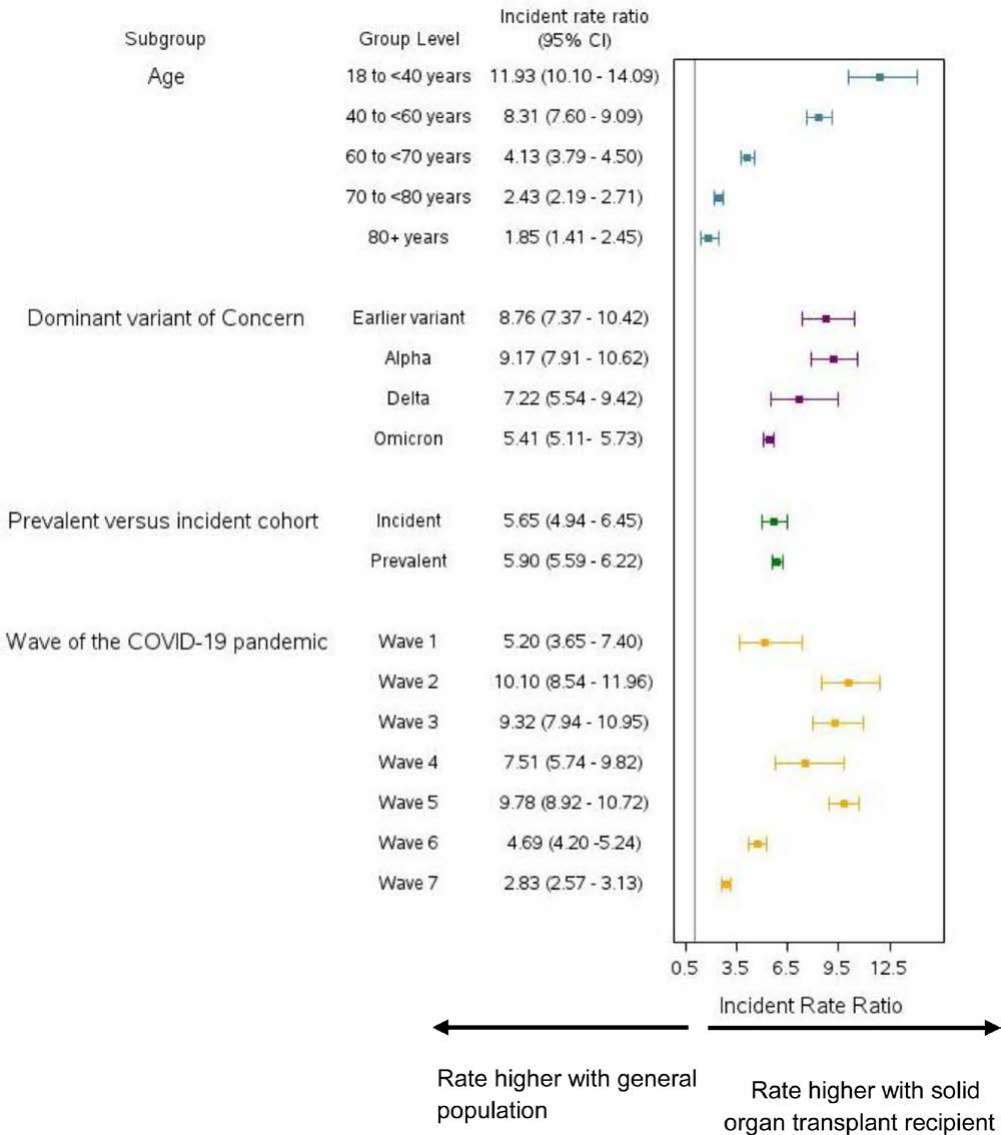
Incidence rate ratios for hospitalization or death with a positive SARS-CoV-2 test for solid organ transplant recipients compared with the general population, presented by subgroups. The horizontal lines indicate 95% CIs. The prevalent cohort is defined as anyone entering the cohort on January 25, 2020; the incident cohort is defined as anyone entering the cohort between January 26, 2020, and September 20 2022. For solid organ transplant recipients in the prevalent cohort the median time since transplant was 7 y (3–13 y). The 7 waves of the COVID-19 pandemic were defined as: wave 1 (January 25, 2020, to August 31, 2020), wave 2 (September 1, 2020, to February 28, 2021), wave 3 (March 1, 2021, to July 31, 2021), wave 4 (August 1, 2021, to December 14, 2021), wave 5 (December 15, 2021, to February 28, 2022), wave 6 (March 1, 2022, to June 18, 2022), and wave 7 (June 19, 2022, to November 30, 2022). CI, confidence interval.

**FIGURE 2.**
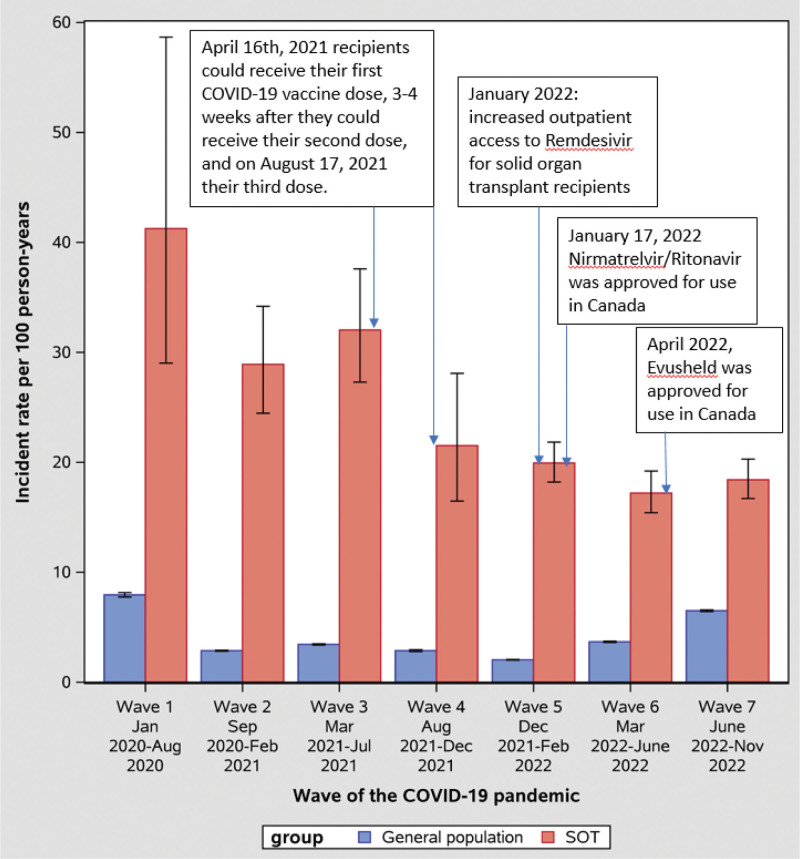
Incidence rate for hospitalization or death with a positive SARS-CoV-2 test for SOT recipients and the general population, presented by wave of the COVID-19 pandemic. Error bars indicate 95% confidence intervals. SOT, solid organ transplant.

Across all prespecified subgroups, we found that the rate of severe COVID-19 was substantially higher in solid organ transplant recipients compared with the general population (Table 4; Figure 1). For example, solid organ transplant recipients aged 18 to <40 y had almost 12 times as high a rate of severe COVID-19 when compared with the general population (IRR, 11.93; 95% CI, 10.10-14.09). As age increased, the IRR decreased, with the lowest IRR observed in the subgroup of individuals aged 80+ y (IRR, 1.85; 95% CI, 1.41-2.45). When examining the rate of severe COVID-19 across pandemic waves, we found that the most recent 2 waves (wave 6: March 1, 2022, to June 18, 2022 and wave 7: June 19, 2022, to November 30, 2022) had the lowest IRR (Figure 2).

**TABLE 4.** Rate of severe COVID-19 (ie, death or hospitalization with a positive SARS-CoV-2) test among solid organ transplant recipients and the general population by age group, dominant variant of concern, prevalent versus incident status, and pandemic wave

Subgroup	Solid organ transplant population^*a*^ (n = 3385)	General population^*a*^ (n = 1 094 440)	Incidence rate ratio (95% CI)
	Rate per 100 person-years (95% CI)	Rate per 100 person-years (95% CI)	
Age, y			
18 to <40	11.79 (9.99-13.91)	0.99 (0.97-1.01)	11.93 (10.10-14.09)
40 to <60	16.45 (15.06-17.97)	1.98 (1.95-2.01)	8.31 (7.60-9.09)
60 to <70	25.29 (23.25-27.51)	6.12 (6.03-6.22)	4.13 (3.79-4.5)
70 to <80	29.11 (26.17-32.38)	11.97 (11.81-12.13)	2.43 (2.18-2.71)
80+	27.34 (20.72-36.07)	14.74 (14.58-14.91)	1.85 (1.41-2.45)
Dominant variant of concern			
Earlier variant/non-variant of concern: before January 17, 2021	30.45 (25.62-36.18)	3.47 (3.42-3.53)	8.76 (7.37-10.42)
Alpha: January 17, 2021, to June 12, 2021	31.46 (27.17-36.42)	3.43 (3.38-3.48)	9.17 (7.91-10.62)
Delta: June 13, 2021, to December 1, 2021	24.9 (19.11-32.43)	3.45 (3.35-3.55)	7.22 (5.54-9.42)
Omicron: December 1, 2021, to November 30, 2022	18.6 (17.57-19.68)	3.44 (3.41-3.47)	5.41 (5.11-5.73)
Entered the study in the prevalent vs incident cohort			
Incident (cohort entry date > January 25, 2020)	29.21 (25.61-33.3)	5.17 (5.08-5.27)	5.66 (4.94-6.44)
Prevalent (cohort entry date January 25, 2020)	19.41 (18.4-20.48)	3.29 (3.27-3.31)	5.9 (5.59-6.22)
Wave of the COVID-19 pandemic			
Wave 1: January 25, 2020, to August 31, 2020	41.25 (29.01-58.66)	7.94 (7.74-8.15)	5.20 (3.65-7.40)
Wave 2: September 1, 2020, to February 28, 2021	28.91 (24.45-34.18)	2.86 (2.81-2.91)	10.10 (8.54-11.96)
Wave 3: March 1, 2021, to July 31, 2021	32.02 (27.29-37.58)	3.44 (3.38-3.49)	9.32 (7.94-10.95)
Wave 4: August 1, 2021, to December 14, 2021	21.51 (16.47-28.09)	2.87 (2.78-2.95)	7.51 (5.74-9.82)
Wave 5: December 15, 2021, to February 28, 2022	19.93 (18.20-21.83)	2.04 (2.01-2.07)	9.78 (8.92-10.72)
Wave 6: March 1, 2022, to June 18, 2022	17.20 (15.40-19.20)	3.67 (3.61-3.73)	4.69 (4.20-5.24)
Wave 7: June 19, 2022, to November 30, 2022	18.41 (16.71-20.28)	6.49 (6.42-6.57)	2.83 (2.57-3.13)

The incidence rate ratio results are also presented graphically in Figure 1.

^*a*^The denominator is restricted to individuals who tested positive for SARS-CoV-2.

CI, confidence interval.

We found that 68.6% of solid organ transplant recipients received at least 1 SARS-CoV-2 test during follow-up, compared with 45.7% in the general population. The mean number of tests was 3.1 ± 5.1 in solid organ transplant recipients compared with 1.5 ± 4.1 in the general population. Among solid organ transplant recipients admitted to the hospital with a positive SARS-CoV-2 test, 28.9% were admitted to the intensive care unit compared with 18.3% in the general population (IRR, 1.60; 95% CI, 1.45-1.75). The median length of hospital stay was 7 d (4–15 d) in solid organ transplant recipients compared with 6 d (3–13 d) in the general population (*P* < 0.001).

When examining SARS-CoV-2 reinfection, we found a similar proportion of solid organ transplant recipients and the general population had a reinfection, 3.8% and 3.3%, respectively.

## DISCUSSION

In this study, we found that despite access to vaccination and the latest therapeutics, solid organ transplant recipients continue to experience a substantially higher rate of hospitalization or death with a positive SARS-CoV-2 test when compared with the general population. Younger solid organ transplant recipients and lung and multiorgan transplant recipients have a particularly high rate of hospitalization or death when compared with the general population. These findings highlight the continued need for strategies to mitigate SARS-CoV-2 exposure and to promote uptake of the COVID-19 vaccine among solid organ transplant recipients.

Our findings are consistent with several previous studies.^5,15,16,23^ For example, a study conducted in England found that compared with an age-matched general population, kidney transplant recipients have a substantially higher risk of death from COVID-19, across COVID-19 pandemic waves, including the Omicron era.^5^ Unlike our study, they found that the Omicron wave did not have the lowest mortality rate. However, their follow-up stopped in March 2022, failing to incorporate more recent pandemic waves (ie, waves 6 and 7 in our study). A study conducted in Korea matched solid organ transplant recipients to nonsolid organ transplant recipients by propensity score and found that solid organ transplant recipients had a significantly higher odds of severe COVID-19 (defined as requirement for respiratory failure treatment such as mechanical ventilation), with an adjusted odds ratio of 18 for lung/heart transplant recipients.^23^

Conversely, Gatti et al^14^ conducted a meta-analysis of 30 studies finding that solid organ transplant recipients did not have a higher risk of mortality from COVID-19 when compared with the general population. However, this was after adjusting for demographic and clinical characteristics and predated the Omicron era. Moreover, none of the included studies were deemed at low risk of bias.^14^ Unlike previous publications, our study provided a comprehensive examination of COVID-19, including all solid organ transplant types, examining multiple COVID-19-associated outcomes (eg, death, mortality, reinfection), included multiple pandemic waves (including Omicron, with data until November 2022), and aimed to elucidate the magnitude of the difference in COVID-19 outcomes between recipients and the general population to guide resource and capacity planning.

Similar to other studies, we found that multiorgan transplant recipients and lung transplant recipients had the highest rate of severe COVID-19 outcomes among solid organ transplant types.^15-17,24^ One potential reason for the higher risk of severe outcomes is because of the need for higher immunosuppression in these groups, with 1 study finding mycophenolic acid and steroids are associated with in an increased COVID-19 hospitalization risk.^25,26^ This highlights that multiorgan and lung transplant recipients are particularly vulnerable populations that might require enhanced protections and perhaps novel therapies to target SARS-CoV-2 infections.

In our study, solid organ transplant recipients with severe COVID-19 were substantially younger than the general population with approximately 3% of recipients versus 30% of the general population being 80 y old or older. Furthermore, we found the rate of severe COVID-19 was particularly high in solid organ transplant recipients between the ages of 18 and 39 y, with recipients having almost 12 times the rate of hospitalization or death when compared with the general population. As age increased, the difference in the rate of severe outcomes between the 2 groups became less pronounced, with the smallest incident rate ratio occurring in the subgroup of individuals aged 80 y old and older. Older age is an established major risk factor for serious COVID-19 illness but our findings highlight that recipients experience serious outcomes at a younger age, likely because of the presence of several other risk factors (eg, comorbidities like diabetes and the use of immunosuppression medications).^26,27^

Across the 7 COVID-19 pandemic waves, we found the lowest incidence rate of severe COVID-19 in solid organ transplant recipients occurred in the last 2 waves (ie, waves 6 and 7), which encompassed the Omicron era. However, we found the risk of severe COVID-19 remained substantially higher than the general population across all waves. Previous studies have found similar results.^23^ For example, Villanego et al^28^ found kidney transplant recipients were less likely to be admitted to the intensive care unit or die during the Omicron wave (follow-up until February 2022) when compared with the Delta wave. Anjan et al^29^ studied 166 solid organ transplant recipients until January 2022 finding higher rates of hospitalization during the Omicron surge but lower mortality. Using survey data, Chiang et al^30^ reported a marked decrease in COVID-19 hospitalization among solid organ transplant recipients when comparing pre-Delta and Delta to Omicron. Factors such as vaccination, access to novel therapeutics, and changes in viral pathogenicity are likely important contributors to the decrease in severe outcomes of COVID-19 seen in more recent pandemic waves.

The continued high risk of severe COVID-19 in the solid organ transplant population is concerning. Even with the availability of vaccinations and therapeutics, we still found that solid organ transplant recipients remain disproportionately impacted by COVID-19. For example, in the most recent pandemic wave (wave 7: June 19, 2022, to November 30, 2022), we found recipients still had almost a 3 times higher rate of severe COVID-19 when compared with the general population. It is important to note that COVID-19 vaccine effectiveness has been found to be lower in solid organ transplant recipients when compared with the general population, likely contributing to the continued heightened risk of severe outcomes.^31,32^ Also of concern is the trend toward reduced vaccine uptake. For example, in Ontario, Canada, 95% of solid organ recipients aged ≥60 y had at least 1 dose of the COVID-19 vaccine but decreased to 72% with a fourth dose.^33^ It is important to note that solid organ transplant recipients still have a higher vaccine uptake than the general population, with only 60% of the general population ≥60 y receiving a fourth dose.^33^

Although therapeutics have been demonstrated to lower the risk of death or hospitalization in solid organ transplant recipients, there are still challenges.^34-36^ For example, the use of Nirmatrelvir/Ritonavir can lead to severe drug-drug interactions with some transplant medications.^34^ In Canada, Nirmatrelvir/Ritonavir was approved for use in January 2022 and in January 2022 outpatient access to Remdesivir in solid organ transplant recipients increased. In April 2022, Tixagevimab/Cilgavimab was approved. Administration of these preventative and therapeutic agents required unique distribution and payment mechanisms coordinated within Ontario and rapid access through transplant hospitals and their vaccination and ambulatory transplant clinics. These substantial efforts were likely only accessed by a proportion of the population at risk.

There are a high number of solid organ transplant recipients being infected with COVID-19, with 22% of our cohort being infected during our study period. It is important to note that this is likely an underestimate, with decreased access to RT-PCR tests starting in December 2021. There is an urgent need to study long-term outcomes of COVID-19 in this patient population. Among people in the general population, numerous studies have found adverse effects on multiple organ systems during the post-acute phase of SARS-CoV-2 infection, including adverse cardiovascular and neurological outcomes.^37-39^

There is also a concern with the adverse health effects associated with individuals having repeated COVID-19 infections. For example, a large cohort study using the Veterans Affair’s US National Healthcare Database found that, when compared with individuals with no SARS-CoV-2 reinfection, individuals with a reinfection had further increases in the risk of death, hospitalization, and various clinical sequalae (eg, cardiovascular, neurological, and mental health disorders) which persisted at 6 mo into the post-acute phase.^40^ In our study, we found that approximately 3% of individuals in both groups were reinfected with COVID-19. However, with the wide circulation of the JN.1 variant and a decrease in mitigation measures, this number is likely much higher now. Somewhat reassuringly, initial research suggests that solid organ transplant recipients reinfected with COVID-19 may have better outcomes in the acute phase (eg, significantly lower hospitalization) and improved immunity compared with those with a first infection.^41,42^ This is another area that needs to be further studied in the solid organ transplant population.

There were several limitations of our work. First, we did not have complete information on prescription drug usage (eg, immunosuppression medications; preventative and therapeutic agents for COVID-19). Second, we did not control for baseline differences (eg, age, comorbidities) between the solid organ transplant population and the general population, with the prevalence of several comorbidities (eg, diabetes) substantially higher in transplant recipients. However, controlling for differences would reduce the degree of disparity between the groups making it seem like the rate of hospitalization or death is more similar, which could potentially result in an incorrect conclusion about resource needs for both populations.^18^ Nevertheless, it would still be beneficial for future research to address the risk of severe COVID-19 after controlling for patient comorbidities. Third, we are likely underestimating the number of SARS-CoV-2 infections because of restrictions on laboratory-based RT-PCR testing starting December 30, 2021, and restrictions to testing in the early pandemic period.^43^ Fourth, we did not have data in our administrative databases on whether oxygen was required during a hospitalization with a positive SARS-CoV-2 test. Lastly, these results do not incorporate the most recent COVID-19 variant (JN.1); initial research suggests the new variant is highly contagious but does not appear to be causing worse outcomes (eg, hospitalizations) in the general population.^44^

In conclusion, this study highlights the continued impact of COVID-19 on solid organ transplant recipients, who have a significantly higher rate of hospitalizations and deaths with a positive SARS-CoV-2 test when compared with the general population. There is an urgent need for strategies to reduce SARS-CoV-2 exposure, increase vaccination uptake, and understand long-term COVID-19 outcomes in the solid organ transplant population.

## Supplementary Material


