# Supplementary material for: Comparison of COVID-19 Hospitalization and Death Between Solid Organ Transplant Recipients and the General Population in Canada, 2020–2022
Source: Transplant Direct. 2024 Jun 26;10(7):e1670. doi: 10.1097/TXD.0000000000001670 (PMC11216672; doi:10.1097/TXD.0000000000001670)

Supporting Information

Table S1. The Reporting of studies Conducted using Observational Routinely-collected health Data RECORD statement

	Item No.	STROBE items	Location in manuscript where items are reported	RECORD items	Location in manuscript where items are reported
**Title and abstract**					
	1	(a) Indicate the study's design with a commonly used term in the title or the abstract (b) Provide in the abstract an informative and balanced summary of what was done and what was found	Abstract	RECORD 1.1: The type of data used should be specified in the title or abstract. When possible, the name of the databases used should be included.<br><br>RECORD 1.2: If applicable, the geographic region and timeframe within which the study took place should be reported in the title or abstract.<br><br>RECORD 1.3: If linkage between databases was conducted for the study, this should be clearly stated in the title or abstract.	Abstract<br><br>Abstract<br><br>Abstract
**Introduction**					
Background rationale	2	Explain the scientific background and rationale for the investigation being reported	Introduction		
Objectives	3	State specific objectives, including any prespecified hypotheses	Introduction		

Methods					
Study Design	4	Present key elements of study design early in the paper	Methods		
Setting	5	Describe the setting, locations, and relevant dates, including periods of recruitment, exposure, follow-up, and data collection	Methods		
Participants	6	*(a) Cohort study* - Give the eligibility criteria, and the sources and methods of selection of participants. Describe methods of follow-up<br><br>*Case-control study* - Give the eligibility criteria, and the sources and methods of case ascertainment and control selection. Give the rationale for the choice of cases and controls<br><br>*Cross-sectional study* - Give the eligibility criteria, and the sources and methods of selection of participants<br><br>*(b) Cohort study* - For matched studies, give matching criteria and number of exposed and unexposed<br><br>*Case-control study* - For matched studies, give matching criteria	Methods	RECORD 6.1: The methods of study population selection (such as codes or algorithms used to identify subjects) should be listed in detail. If this is not possible, an explanation should be provided.<br><br>RECORD 6.2: Any validation studies of the codes or algorithms used to select the population should be referenced. If validation was conducted for this study and not published elsewhere, detailed methods and results should be provided.<br><br>RECORD 6.3: If the study involved linkage of databases, consider use of a flow diagram or other graphical display to demonstrate the data linkage process, including the number of individuals with linked data at each stage.	Table S2<br><br>N/A<br><br>Figure S1, S2

		and the number of controls per case	N/A		
Variables	7	Clearly define all outcomes, exposures, predictors, potential confounders, and effect modifiers. Give diagnostic criteria, if applicable.	Methods	RECORD 7.1: A complete list of codes and algorithms used to classify exposures, outcomes, confounders, and effect modifiers should be provided. If these cannot be reported, an explanation should be provided.	Table S2
Data sources/ measurement	8	For each variable of interest, give sources of data and details of methods of assessment (measurement).<br><br>Describe comparability of assessment methods if there is more than one group	Table S2		
Bias	9	Describe any efforts to address potential sources of bias	Methods		
Study size	10	Explain how the study size was arrived at	Figure S1, S2		
Quantitative variables	11	Explain how quantitative variables were handled in the analyses. If applicable, describe which groupings were chosen, and why	Methods		
Statistical methods	12	(a) Describe all statistical methods, including those used to control for confounding	Methods		

		(b) Describe any methods used to examine subgroups and interactions<br><br>(c) Explain how missing data were addressed<br><br>(d) *Cohort study* - If applicable, explain how loss to follow-up was addressed<br><br>*Case-control study* - If applicable, explain how matching of cases and controls was addressed<br><br>*Cross-sectional study* - If applicable, describe analytical methods taking account of sampling strategy<br><br>(e) Describe any sensitivity analyses	Methods<br><br>Methods<br><br>Methods<br><br>n/a		
Data access and cleaning methods		N/A		RECORD 12.1: Authors should describe the extent to which the investigators had access to the database population used to create the study population.<br><br>RECORD 12.2: Authors should provide information on the data cleaning methods used in the study.	Methods<br><br>Figure S1, S2
Linkage		N/A		RECORD 12.3: State whether the study included person-level, institutional-level, or other data linkage across two or more databases. The methods of	Methods

				linkage and methods of linkage quality evaluation should be provided.	
**Results**					
Participants	13	(a) Report the numbers of individuals at each stage of the study (*e.g.*, numbers potentially eligible, examined for eligibility, confirmed eligible, included in the study, completing follow-up, and analysed)<br><br>(b) Give reasons for non-participation at each stage.<br><br>(c) Consider use of a flow diagram	Figure S1, S2<br><br>Not applicable<br><br>Figure S1, S2	RECORD 13.1: Describe in detail the selection of the persons included in the study (*i.e.,* study population selection) including filtering based on data quality, data availability and linkage. The selection of included persons can be described in the text and/or by means of the study flow diagram.	Methods, Figure S1, S2
Descriptive data	14	(a) Give characteristics of study participants (*e.g.*, demographic, clinical, social) and information on exposures and potential confounders<br><br>(b) Indicate the number of participants with missing data for each variable of interest<br><br>(c) *Cohort study* - summarise follow-up time (*e.g.*, average and total amount)	Results, Table 1<br><br>Table 1<br><br>Results		
Outcome data	15	*Cohort study* - Report numbers of outcome events or summary measures over time	Results, Table 2		

		Case-control study - Report numbers in each exposure category, or summary measures of exposure<br><br>*Cross-sectional study* - Report numbers of outcome events or summary measures			
Main results	16	(a) Give unadjusted estimates and, if applicable, confounder-adjusted estimates and their precision (e.g., 95% confidence interval). Make clear which confounders were adjusted for and why they were included<br><br>(b) Report category boundaries when continuous variables were categorized<br><br>(c) If relevant, consider translating estimates of relative risk into absolute risk for a meaningful time period	Table 2, Table 4, Figures 1 and 2<br><br>Table 4, Figure 1<br><br>Not applicable		
Other analyses	17	Report other analyses done—e.g., analyses of subgroups and interactions, and sensitivity analyses	Methods, Results, Table 4, Figures 1 and 2		
**Discussion**					
Key results	18	Summarise key results with reference to study objectives	Discussion		

Limitations	19	Discuss limitations of the study, taking into account sources of potential bias or imprecision. Discuss both direction and magnitude of any potential bias	Discussion	RECORD 19.1: Discuss the implications of using data that were not created or collected to answer the specific research question(s). Include discussion of misclassification bias, unmeasured confounding, missing data, and changing eligibility over time, as they pertain to the study being reported.	Discussion
Interpretation	20	Give a cautious overall interpretation of results considering objectives, limitations, multiplicity of analyses, results from similar studies, and other relevant evidence	Discussion		
Generalisability	21	Discuss the generalisability (external validity) of the study results	Discussion		
**Other Information**					
Funding	22	Give the source of funding and the role of the funders for the present study and, if applicable, for the original study on which the present article is based	Acknowledgements		
Accessibility of protocol, raw data, and programming code		N/A		RECORD 22.1: Authors should provide information on how to access any supplemental information such as the study protocol, raw data, or programming code.	The dataset from this study is held securely in coded form at ICES. While legal data sharing agreements between ICES and data providers (e.g., healthcare organizations and government) prohibit ICES

					from making the dataset publicly available, access may be granted to those who meet pre-specified criteria for confidential access, available at www.ices.on.ca/DAS (email: das@ices.on.ca). The full dataset creation plan and underlying analytic code are available from the authors upon request, understanding that the computer programs may rely upon coding templates or macros that are unique to ICES and are therefore either inaccessible or may require modification.

Reference: Benchimol EI, Smeeth L, Guttmann A, Harron K, Moher D, Petersen I, Sørensen HT, von Elm E, Langan SM, the RECORD Working Committee. The REporting of studies Conducted using Observational Routinely-collected health Data (RECORD) Statement. *PLoS Medicine* 2015 Oct 6;12(10):e1001885. Checklist is protected under Creative Commons Attribution (CC BY) license.

Table S2: Coding definition for inclusion, exclusion criteria, exposure, and outcomes

Characteristic	Database	Codes
**Solid organ transplant recipient inclusion criteria**		
Recipients of a solid organ transplant	CORR<br><br>OHIP	TREATMENT_CODE=171<br>TREATMENT_DATE<br>TRANSPLANTED_ORGAN_TYPE[1-3]: 10-12, 18-23, 29-30, 40-43, 48-55, 60, 90, 99<br>Feecode: S435, S434, S197, S294, S295, S266, R870, M155, M156, S308, S202, E807
Alive as of index date	RPDB	dthdate
**Solid organ transplant recipient exclusion criteria**		
Missing or invalid ICES key number, missing or invalid age (>105), missing or invalid sex, non-Ontario resident, age <18	RPDB	
Graft failure (only applicable in kidney transplant recipients and defined by post-transplant maintenance dialysis)	 OHIP<br><br>CIHI-DAD	Look forward from the date of transplant for first evidence of two OHIP or CIHI chronic dialysis codes separated by at least ≥ 90 days but < 150 days<br>Feecode: R849, G323, G325, G326, G860, G862, G865, G863, G866, G330, G331, G332*, G333, G861, G082, G083, G085, G090, G091, G092, G093, G094, G095, G096, G294, G295, G864, H540, H740<br>*G332= PD-Chronic (up to 49hrs) from 1992 to 1998, this is a recycled code and from 2008 on it captures capsule endoscopy<br>CCP: 5195, 6698<br>CCI: 1PZ21

No evidence of Ontario Health Insurance Plan eligibility	RPDB	Eligon
Positive COVID-19 test prior to January 25, 2020	C19INTGR	COVIDRESULT=”P”
A COVID-19 positive test result in the 90 days prior to transplant.	C19INTGR	COVIDRESULT=”P”<br>Note: This exclusion is only applicable to the incident cohort
**Categorization of solid organ transplant types**		
Kidney-only transplant recipient	CORR<br><br><br>OHIP	Treatment_Code: 171<br>Treatment_Date<br>Transplanted_Organ_Type_Code: 10, 11, 12, 18, 19<br><br>Feecode: S435, S434<br>Excluded multiorgan transplants:<br>Feecode: S294, S295, S202, E807, M155, M156, R870, S308, S197, S266
Liver-only transplant	CORR<br><br><br><br>OHIP	Treatment_Code: 171<br>Treatment_Date<br>Transplanted_Organ_Type_Code: 20, 21, 22, 23, 29<br><br><br>Feecode: S294, S295, S266<br>Excluded multiorgan transplants:<br>Feecode: S435, S434, S202, E807, M155, M156, R870, S308, S197
Heart-only transplant	CORR<br><br><br>OHIP	Treatment_Code: 171<br>Treatment_Date<br>Transplanted_Organ_Type_Code: 30<br><br>Feecode: R870<br><br>Excluded multiorgan transplants:

		Feecode: S435, S434, S202, E807, M155, M156, S308, S294, S295, S197, S266
Lung-only transplant	CORR<br><br><br><br><br>OHIP	Treatment_Code: 171<br>Treatment_Date<br>Transplanted_Organ_Type_Code: 40, 41, 42, 48, 49<br><br><br>Feecode: M155, M156<br>Excluded multiorgan transplants:<br>Feecode: S435, S434, S202, E807, S308, S294, S295, R870, S197, S266
Any pancreas transplant (can be multiorgan)	CORR<br><br><br><br>OHIP	Treatment_Code: 171<br>Treatment_Date<br>Transplanted_Organ_Type_Code: 50, 51, 52, 53, 54, 55<br><br>Feecode: S308
Multiorgan transplant (excluding pancreas)	CORR<br><br><br><br><br>OHIP	Treatment_Code: 171<br>Treatment_Date<br>Transplanted_Organ_Type_Code: 10-12, 18-23, 29-30, 40-43, 48-49, 60, 90, 99<br><br><br>Feecode: S435, S434, S197, S294, S295, R870, M155, M156, S202, E807, S266
**General population-inclusion criteria**		
Alive as of index date	RPDB	dthdate
**General population-exclusion criteria**		
Missing or invalid ICES key number, missing or invalid age (>105), missing or invalid sex, non-Ontario resident, age <18	RPDB	
No evidence of Ontario Health Insurance Plan eligibility	RPDB	Eligon

Positive COVID-19 test prior to January 25, 2020	C19INTGR	COVIDRESULT=”P”
A COVID-19 positive test result in the 90 days prior to index date	C19INTGR	COVIDRESULT=”P”<br>Note: This exclusion is only applicable to the incident cohort
Received a previous solid organ transplant or in solid organ transplant cohort	CORR. RECIPIENT_TREATMENT<br><br>OHIP	TREATMENT_CODE=171<br>TRANSPLANTED_ORGAN_TYPE[1-3]: 10, 11, 12, 18, 19, 20, 21, 22, 23, 29, 30, 40, 41, 42, 48, 49, 43, 50, 51, 52, 53, 54, 55, 60, 90, 99<br><br>FEECODE: S435, S434, S197, S294, S295, S266, R870, M155, M156, S308, S202, E807
**Demographics**		
Age, Sex	RPDB	
Neighborhood income quintile	RPDB	
Rurality	RPBD	
Long term care residence	ODB<br>CCRS	LTC=1 Within 120 days prior to index date (ODB)<br>CCRS anytime before index date
**Comorbidities**		
Charlson Comorbidity Index	CIHI	ICD-10
Congestive Heart Failure	CHF	Limited to ages 40+ and defined as 1 hospitalization OR 1 physician visit/emergency department visit followed by 1 hospitalization/emergency department visit/physician visit within 1-year for congestive heart failure.
Hypertension	HYPER	Defined as one hospital admission captured in DAD/SDS that has a hypertension diagnosis (ICD 10: I10, I11, I12, I13, I15) OR an OHIP diagnosis code (401, 402, 403, 404, or 405) with a hypertension diagnosis followed by another OHIP diagnosis code for hypertension or a hospital admission with a hypertension diagnosis within 2 years

Diabetes	Ontario Diabetes Database	Defined as 2 OHIP diagnosis code claims (250) or 1 Ontario Drug Benefit drug claim for diabetes or 1 hospital admission within 1 year.
Dementia	Dementia database	Limited to adults aged 40 to 110 years and defined as: ≥1 Hosp (DAD/SDS) or ≥1 ODB claim for cholinesterase inhibitors or ≥3 OHIP at least 30 days apart in a two-year period
Stroke (excluding transient ischemic attack)	DAD<br><br>OHIP	Defined as a hospitalization or 2 physician billings within a one-year period<br>ICD-10: H341, I60, I600, I601, I602, I603, I604, I605, I606, I607, I608, I609, I61, I610, I611, I612, I613, I614, I615, I616, I618, I619, I630, I631, I632, I633, I634, I635, I638, I639, I64<br><br>OHIP DXCODE: 432, 436
Myocardial Infarction	DAD	ICD-10: I21, I22
Chronic respiratory disease	ASTHMA<br><br>COPD	Evidence of asthma defined as ≥ 1 hospitalization (DAD) or 2 physician billing claims (OHIP) for asthma in a two-year period<br><br>OR<br><br>Evidence of chronic obstructive pulmonary disease defined as ≥1 hospitalization (DAD/SDS) or ≥1 physician billing (OHIP)
Chronic kidney disease	DAD/SDS<br><br>NACRS<br><br>OHIP	1 hospitalization/emergency department visit or 3 physician diagnosis codes in 1 year of any of the following codes:<br><br>ICD-10: E102, E112, E132, E142, I12, I13, N08, N18, N19<br><br>OHIP DXCODE: 403, 585

Major cancer	DAD/SDS<br><br>OHIP	ICD10: 971, 980, 982, 984, 985, 986, 987, 988, 989, 990, 991, 993, C15, C18, C19, C20, C22, C25, C34, C50, C56, C61, C82, C83, C85, C91, C92, C93, C94, C95, D00, D011, D012, D022, D05, D075<br><br>OHIP DXCODE: 150, 154, 155, 157, 162, 174, 175, 183, 185, 203, 204, 205, 206, 207, 208
Chronic liver disease	DAD/SDS<br>NACRS<br>OHIP	ICD10: B16, B17, B18, B19, B942, E830, E831, , I85, K70, K713, K714, K715, K717, K721, K729, K73, K74, K76, K77, R160, R162, R17, R18, Z225<br><br>OHIP DXCODE: 571, 573, 700, 701, 703, 704, 706, 707, 708, 709<br><br>OHIP FEECODE: Z551, Z554
**COVID-19 Specific Characteristics**		
COVID-19 Vaccine status	COVaxON	DOSE1_DATE_C<br>DOSE2_DATE_C<br>DOSE3_DATE_C<br>DOSE4_DATE_C<br>DOSE5_DATE_C<br>DOSE6_DATE_C
Time since last vaccination	C19INTGR<br><br>COVAXon	COVIDRESULT=”P”<br><br>Select the positive COVID test associated with the hospitalization or death.<br><br>Select the Dose_Date_C closest to the above positive COVID-19 positive test
Dominant Variant of Concern		1. Earlier variant/non-VOC: Prior to January 17, 2021;<br><br>2. Alpha: January 17, 2021 to Jun 12, 2021<br><br>3. Delta: June 13, 2021 to December 1, 2021

		4. Omicron: December 1, 2021 onwards<br><br>These dates were determined based on publicly available information.<br><br>This information relates to when the individual tested positive and then we placed them in the appropriate time-frame based on when they tested positive.
Prior COVID-19 Infection	C19INTGR	COVIDRESULT="P"<br><br>OBSERVATIONDATE value >90 days prior to index date
**Outcomes**		
Hospitalization or death with a positive SARS-CoV-2 test	**1.Death**<br><br>CCM<br><br>C19INTGR<br><br>RPDB<br><br>Or<br><br>**2. Hospitalization**<br><br>CCM<br><br>C19INTGR<br><br>DAD/SDS	Defined as either a death or hospitalization associated with COVID-19. Our total numbers are based on a composite of CCM AND RPDB/DAD. An individual can only be counted once.<br><br>**DEATH**: CCM: OUTCOME= "FATAL" OR RPDB: DTHDATE,<br><br>A death in RPDB is defined as a positive COVID-19 test occurring in the 30 days prior to death. Date of death recorded in CCM or RPDB is considered the date the outcome occurred. The COVID-19 positive test had to occur on or after the index date.<br><br>**HOSPITALIZATION:** A hospitalization with COVID-19 in DAD is defined as a positive test which must have occurred within 14 days prior or 3 days after admission. The hospital admission date will be the date used to determine the time frame of the outcome. Both the COVID-19 test and hospitalization had to occur after the index date.<br><br>For CCM: HOSPITALIZED "YES" OR ICU="YES"
Hospitalization with a positive SARS-CoV-2 test	CCM<br>C19INTGR<br>DAD/SDS	Same hospitalization definition as above.

Death with a positive SARS-CoV-2 test	CCM<br>C19INTGR<br>RPDB	Same death definition as above.
Length of stay for a hospital admission with a positive COVID-19 test	CCM<br><br>DAD/SDS	Length of stay is calculated by calculating the time between the HOSP_ADMDATE and the HOSP_DDATE if captured in CCM or the ADMDATE and DDATE if captured in DAD/SDS.<br><br>If an individual was not discharged from the hospital prior to the end of follow-up, the calculation stops on the last day of follow-up.
Admission to the intensive care unit with a positive SARS-CoV-2 test test	CCM<br><br>DAD/SDS	Evidence of an ICU stay within hospitalization (i.e., ICU stay falls within the admdate and ddate of the COVID-related hospitalization).<br><br>First look in the CCM database, look directly at the ICU=”YES”.<br><br>Then look in DAD/SDS. All non-missing scuadmdate1’s are evidence of an ICU admission. Report the unique ICU admissions combining CCM and DAD/SDS.
SARS-CoV-2 infection	C19INTGR	COVIDRESULT=”P”<br><br>Includes both symptomatic and asymptomatic infection (i.e., any positive test result). Observation date is the specimen collection date.<br><br>**Note**: Any individual who was counted in the primary outcome (i.e., had a hospitalization or death with a positive SARS-CoV-2 test recorded in CCM would also be included in this definition).
SARS-CoV-2 test	C19INTGR	COVIDTEST=”T”<br><br>For this outcome only the only censoring events was end of follow-up or death. For example, a patient could be hospitalized for COVID-19 and then be released and then go on to have more COVID-19 tests during the study period. Each subsequent COVID-19 test is counted in this outcome.

Reinfection	C19INTGR	Evidence of a positive PCR test >90d after initial COVID-19 infection.<br><br>Note: The denominator was restricted to individuals who had an initial infection AND were still alive at 90 days after their initial infection.
**Censoring Event**		
Non-COVID death	CCM<br><br>C19INTGR<br><br>RPDB	Defined as a death not associated with COVID-19. CCM: OUTCOME= "FATAL"<br><br>OR<br><br>A death with COVID-19 in RPDB is defined as a positive COVID-19 test occurring in the 30 days prior. The COVID-19 positive test had to occur on or after the index date. If an individual has a death that meets the CCM or RPDB criteria they will not be counted as a censoring event.

Abbreviations: CCI, Canadian Classification of Health Interventions; CCM, Case and Contact Management System; CCP, Canadian Classification of Diagnostic, Therapeutic and Surgical Procedures; CCRS, Continuing Care Reporting System; CIHI, Canadian Institute for Health Information; COPD, Chronic Obstructive Pulmonary Disease; CORR, Canadian Organ Replacement Register; DAD, Discharge Abstract Database; ICD, International Classification of Diseases; ODB, Ontario Drug Benefit; OHIP, Ontario Health Insurance Plan; PCCF, Postal Code Conversion File; RPDB, Registered Persons Database; SDS, Same Day Surgery
C19INTGR, COVID-19 Integrated Testing Data

Figure S1. Cohort selection for solid organ transplant recipients

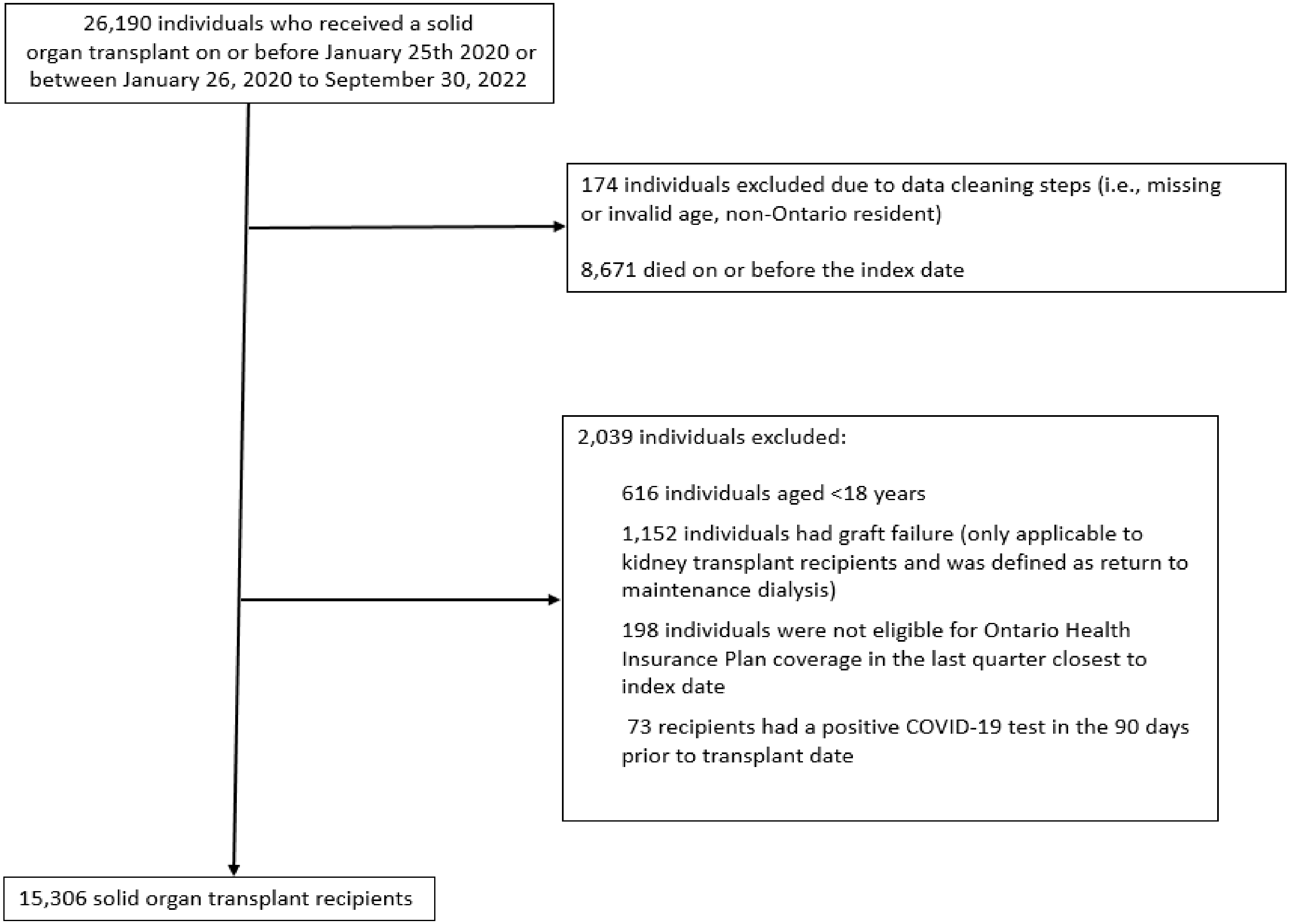

Figure S2. Cohort selection for the general population

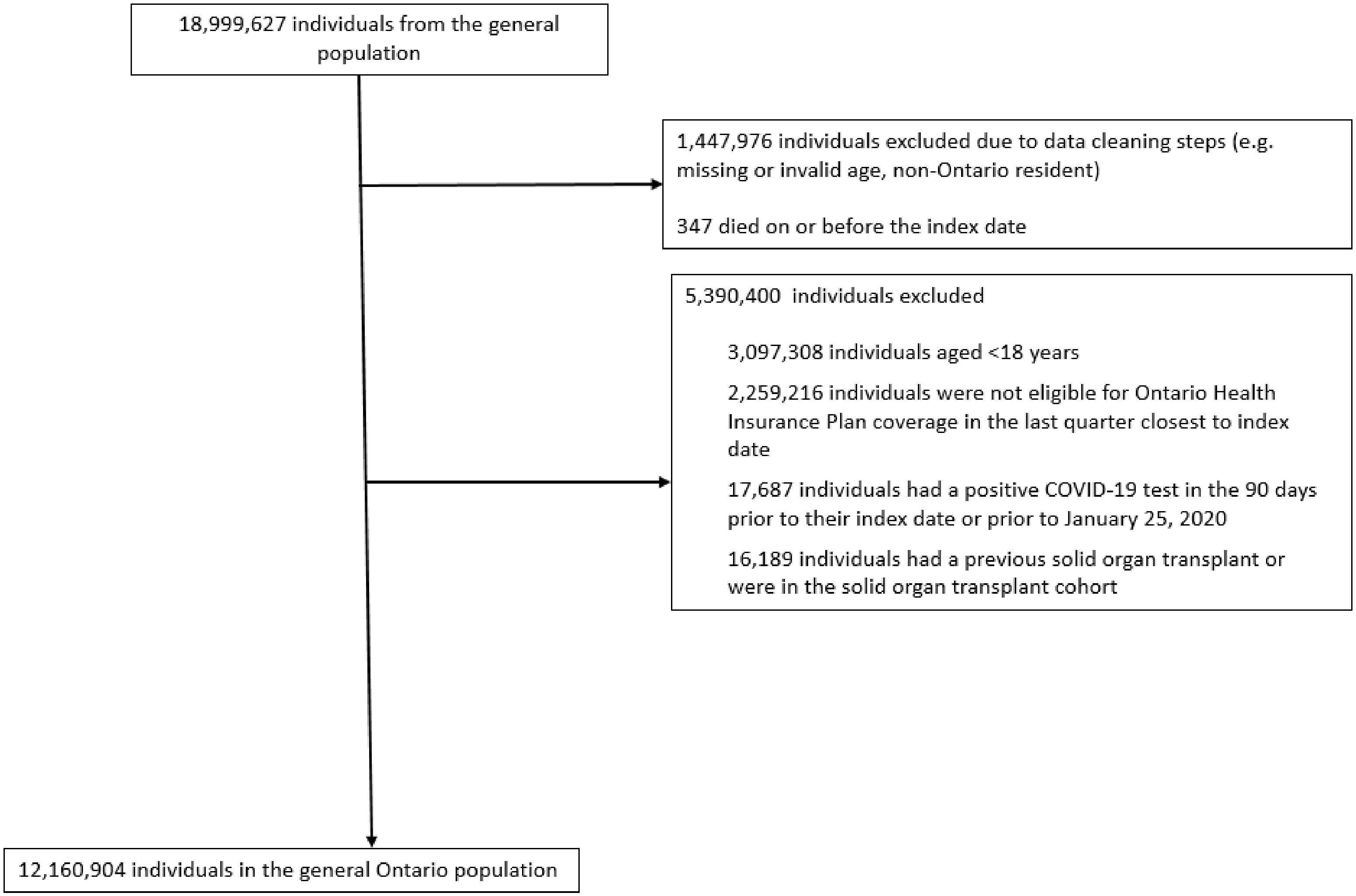

Supplement: Supplementary file 1 [file txd-10-e1670-s001.pdf]
